# Construction of *Candida albicans* pRB895‐ *SAP2* ‐SC5314 With *SAP2* High Expression and Its Effects on Adhesion

**DOI:** 10.1002/jcla.25144

**Published:** 2024-12-27

**Authors:** Lan Xue, Lu Yang, Bingqian Zhao, Wenli Feng, Jing Yang, Yan Ma

**Affiliations:** ^1^ The Department of Dermatovenereology The Second Hospital of Shanxi Medical University Taiyuan Shanxi China

**Keywords:** *C. albicans*, magnolol, pRB895‐*SAP2*‐SC5314 strain, *SAP2*, *SAP2*adhesion

## Abstract

**Background:**

*SAP2* is closely associated with the pathogenicity and drug resistance of *Candida albicans* ( *C. albicans* ). Our study aimed to construct *C. albicans* with *SAP2* overexpression (pRB895‐*SAP2*‐SC5314) to explore the influence of *SAP2* on the adhesion of *C. albicans* and predict the interaction between magnolol and Sap2 by molecular docking.

**Methods:**

The recombinant plasmid pRB895‐*SAP2* with high *SAP2* expression was prepared using a plasmid extraction kit and transformed into *C. albicans* strain SC5314 using an improved lithium acetate conversion method to construct PRB895‐*SAP2*‐SC5314. Quantitative reverse transcription polymerase chain reaction (qRT‐PCR) was performed to detect the expression of adhesion‐related genes in the different strains. Molecular docking and visual analysis of magnolol and Sap2 were performed using the CB‐DOCK2 platform.

**Results:**

Compared with the control SC5341 and SC5341 transfected with pRB895, *SAP2* expression was significantly higher in the pRB895‐*SAP2*‐SC5314 strain (*p* < 0.05). Based on the sequencing and mapping results, the pRB895‐*SAP2*‐SC5314 strain was successfully prepared. *SAP2* overexpression significantly downregulated *ALS1* expression (*p* < 0.05), whereas *ALS3*, *TEC1*, *HOG1*, *PHR1*, and *TUP1* expression was downregulated in *C. albicans* (*p* < 0.05). The optimal docking result for magnolol and Sap2 protein was −8.1 kcal/mol of vina score, which was considered good docking.

**Conclusions:**

*SAP2* overexpression may strengthen the adhesion and pathogenicity of *C. albicans* , and magnolol may act as an Sap2 inhibitor that affects the adhesion of *C. albicans* .

## Graphical abstract

Our research successfully established the *C. albicans* strain with *SAP2* overexpression (pRB895‐*SAP2*‐SC5314) using an improved lithium acetate conversion method. *SAP2* overexpression may strengthen the pathogenicity of *C. albicans* through regulating the expression of adhesion‐related genes.

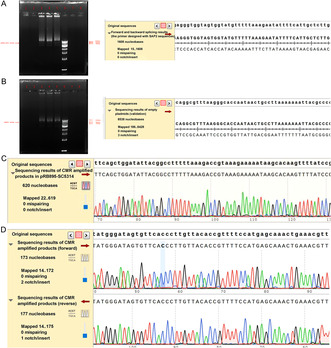

## 1 Introduction

Invasive candidiasis (IC) is a common fungal infectious disease with an annual incidence of about 2–14/100,000 people worldwide and a mortality rate of 40%–55%, which poses a significant threat to patient survival [1]. Among them, *Candida albicans* is the most common pathogen, and the World Health Organization reports that *C. albicans* infection accounts for 50%–70% of all IC cases [2]. Traditional antifungal drugs include azole drugs (fluconazole, itraconazole, and voriconazole) and echinocandins (anidulafungin, caspofungin, and micafungin). Although these drugs have shown strong antifungal activity in clinical applications, their use is usually associated with significant toxic side effects on the liver and kidneys. Additionally, the widespread use of these drugs has led to increased drug resistance rate. For instance, the global average resistance rate of fluconazole and echinocandins are approximately 3.9%, with significant regional differences [3], and approximately 3%–10%, respectively [4]. These factors lead to a high rate of treatment failure and limit the variety of effective drugs available in clinical practice. Therefore, there is an urgent need to identify novel antifungal targets and develop safer and more efficient new antifungal drugs.

The adhesion and invasion of the mucosal barrier function by *C. albicans* are the prerequisites for disseminated candidiasis [5]. The ability to adhere and invade enables *C. albicans* to establish infection in the host and promotes the spread and exacerbation of the infection [6]. Numerous studies have shown that extracellular hydrolytic enzymes secreted by *C. albicans* , such as proteases, lipases, and phospholipases, play important roles in host adhesion, especially the secreted aspartyl protease (Saps) family [7, 8]. In the Saps family, Sap2 is the most highly expressed, destructive, and pathogenic protease and contributes to the pathogenicity and drug resistance of *C. albicans* [9]. Our previous studies have found that *SAP2* can affect the formation of biofilms. In both planktonic and biofilm states, *SAP2* can play a synergistic role with *ERG11*, *GCN4*, and *CAP1* in conferring resistance to azole drugs in *C. albicans* [10, 11, 12]. These results further demonstrate that S*AP2* contributes to virulence, pathogenicity, and itraconazole resistance of *C. albicans* and is a potential biomarker for evaluating the virulence and resistance of strains. Current research on the proteolytic activity of *C. albicans* at home and abroad mainly focuses on *SAP2*. *SAP2* has diverse substrates and complex regulatory mechanisms; therefore, it is challenging to fully understand its unique biological characteristics from a single perspective [13]. Moreover, the expression of *SAP2* is regulated by both the self and external environment (host environment, nutritional status, and other external factors) [14], which increases the difficulty of exploring its specific roles in the pathogenesis of *C. albicans* . Given the increasing drug resistance rate of *C. albicans* , it is important to accurately understand its pathogenic mechanisms.

The initial stage of adhesion is a key step in infection, which helps *C. albicans* colonization and enhances pathogenicity by resisting attacks from the host immune system and the action of antifungal drugs [5]. The core of the adhesion process is the expression of adhesion‐related genes, and the activation and expression of these genes directly determines the adhesion ability of *C. albicans* [15]. *ALS1*, *ALS3*, *HOG1*, *PHR1*, *TUP1*, and *TEC1* are all related to adhesion; adhesion proteins encoded by these genes can bind to host cell receptors, allowing pathogens to firmly adhere to host tissues and effectively avoid recognition and clearance by host immune cells, thereby evading host immune defense [16, 17]. The regulation of adhesion‐related genes is affected by various factors such as environmental conditions and host immune response, which exhibit complicated regulatory mechanisms. However, the regulatory roles of *SAP2* and adhesion‐related genes in *C. albicans* remain unclear.

Magnolol is a natural compound extracted from the traditional Chinese medicine *Magnoliae officinalis* cortex and has been proven to have various biological activities, including anti‐inflammatory, antioxidant, antitumor, and antibacterial activities [18]. A previous study showed that magnolol could suppress adhesion, yeast‐to‐mycelium transition, and biofilm formation in *C. albicans* , and has potential anti‐ *C. albicans* infection [19]; however, the specific molecular mechanism is not fully understood.

Therefore, in this study, the strain pRB895‐*SAP2*‐SC5314 with high *SAP2* expression was constructed to further investigate its regulatory roles in *C. albicans* adhesion, providing new ideas for exploring the pathogenic mechanisms of *C. albicans* . By using molecular docking the binding process of magnolol and *SAP2* receptors were mimicked and key binding sites with high affinity for magnolol in SAP2 receptors and key amino acid residues in ligand‐receptor interactions were identified to promote structure‐based drug design and develop more specific and efficient *SAP2* inhibitors, thereby providing scientific theoretical support for the development of a new generation of antifungal drugs.

## 2 Materials and Methods

### 2.1 Experimental Strains and Reagents

The standard strain of *C. albicans* SC5314 was purchased from the Fungi Center of Pathogenic Microorganism (Virus) Species Preservation Center, Chinese Academy of Medical Sciences, and the Department of Fungi, Institute of Dermatology, Chinese Academy of Medical Sciences. The empty plasmid pRB895 and high expression recombinant plasmid pRB895‐*SAP2* were both obtained from Puruting Biotechnology Co., LTD (Beijing, China); as well as receptive *Escherichia coli* DH5α and Salmon sperm DNA (10 mg/mL) were acquired from Solaibao Technology Co., LTD (Beijing, China). Additionally, selective nutrient deficiency medium SD‐ura‐met‐cys, endotoxin‐free plasmid extraction kit (centrifugal column), Tris‐EDTA buffer (10X TE, pH 7.4), TB Green Premix Ex TaqII, and PrimeScript RT Master Mix were provided by Beijing Pankino Technology Co., LTD (Beijing, China), Tiangen Biochemical Technology (Beijing, China), Beijing Regan Biotechnology Co., LTD (Beijing, China), Takara Bio Inc. (Kyoto, Japan), and Takara Bio Inc., respectively.

### 2.2 Transformation, Isolation, and Identification of pRB895 and pRB895‐ *SAP2* Plasmids

The receptive *E. coli* DH5α stored at −70°C (100 μL) was thawed on ice, and then 10 μL pRB895‐*SAP2* plasmids were added. After gentke mixing, the sample was placed on ice for 30 min. It was then subjected to heat shock at 42°C for 70 s, immediately placed in ice for 2 min, and then added to 900 μL pre‐heated (37°C) Luria‐Bertani (LB) medium without chloramphenicol. After culturing at 180 rpm for 60 min at 37°C, the mixture was centrifuged at 1000 rpm/min for 10 min, and the bacteria was re‐suspended in 50 μL medium. Afterwards, the fungal suspension was coated in the LB agar medium supplemented with 100 μg/mL chloramphenicol and incubated in a constant temperature incubator at 37°C for 12 h.

Plasmids were extracted using an endotoxin‐free plasmid extraction kit (centrifugal column) according to the manufacturer's instructions. Briefly, six fresh single *E. coli* DH5α colonies in the aforementioned medium were selected and inoculated into six centrifuge tubes containing 4 mL LB liquid medium (containing 100 μg/mL chloramphenicol); and incubated at 37°C with 300 rpm for 12 h. After centrifuging at 12000 rpm at 4°C for 30 s, the supernatant was discarded, and the sediments were resuspended with 375 μL STE solution. After centrifuging at 12000 rpm at 4°C for 30 s, the sediments were added to 100 μL pre‐cooled solution I, followed by 200 μL solution II. After quickly mixing five times, the fungal solution was placed on ice, and added to 150 μL pre‐cooled solution III. After placing on ice for 5 min, the fungal solution was centrifuged at 12000 rpm at 4°C for 5 min, the supernatant was added with the equal volume of phenol: chloroform (1:1). After centrifugation at 12000 rpm at 4°C for 5 min, the supernatant was mixed with twice its volume of anhydrous ethanol and placed at room temperature for 5 min to precipitate the plasmids. After centrifuging at 12000 rpm at 4°C for 2 min, the supernatant was discarded, and the sediments were washed with 1 mL pre‐cooled 70% ethanol. After centrifugation, the supernatant was discarded and the tube was dried at room temperature for 20 min. Then, 50 μL deionized water containing DNA‐free RNase (20 μg/mL) was added to dissolve the plasmids, and stored at −20°C for subsequent use.

The extracted plasmids (10 μL) were employed for band analysis by 0.7% agarose gel electrophoresis, as well as used for sequencing and identification according to the *SAP2* sequences and specific primers provided by Puruting Biotechnology Co., LTD. The total length of *SAP2* was 1197 bp, and the primers (Table 1) were synthesized and provided by General Biological Co., LTD (Anhui, China). Snapgene software was used to compare and analyze whether the sequencing results of the isolated plasmids were consistent with the *SAP2* gene sequences provided by Puruting Biotechnology Co. Ltd.

**TABLE 1:** The sequences of all primers.

Primer	Sequences (5′‐3′)
*SAP2*	F: AATACGACTCACTATAGG
	R: AGAAAGTATAGGAACTTC
*CMR*‐1	F: TTCTTGCCCGCCTGATGAAT
	R: ACCGTAACACGCCACATCTT
*CMR*‐2	F: TCACTGGATATACCACCGTT
	R: TTACGCCCCGCCCTGCCACT
*ACT1*	F: ACTACCATGTTCCCAGGTATTG
	R: CCACCAATCCAGACAGAGTATT
*ALS1*	F: ACATACGATGGCTCTGGTTC
	R: TGAACTAGATCAAGCCAAAAAGGTG
*ALS3*	F: CCTCAATTCAAGGGAGGGGG
	R: CACGGGCGACGAAAGAGAT
*HOG1*	F: TCTCCATCTGCAGACATTTTCTT
	R: GTCGTCTTTGAAAACATACACCGT
*PHR1*	F: GTGGCAAATGTGGCCAATGA
	R: ACTTAGAAAAGCCGGGCTGC
*TUP1*	F: CCGTCAACATCGCCGTAAGT
	R: GAGATGGACGAGTTGGTGGG
*TEC1*	F: AGGAGTAGCTTGCGACATCA
	R: ACGTGGTTGCTGTCAATTCC

An appropriate amount of *E. coli* solution containing highly expressed recombinant plasmids pRB895‐*SAP2* was cultured in 300 mL LB liquid medium supplemented with 100 μg/mL chloramphenicol at 37°C for 12 h until the medium became cloudy. Then, a large amount of recombinant plasmids pRB895‐*SAP2* with high *SAP2* expression were extracted, and stored at −20°C. Similarly, a sufficient amount of the empty plasmid pRB895 was obtained and used as an experimental control.

### 2.3 Construction of the pRB895‐ *SAP2* ‐SC5314 Strain

The standard strain of *C. albicans* SC5314 (1 × 10^8^ Colony‐Forming Units (CFU)/ mL) was resuspended in 1 mL pre‐cooled 1X TELiAc (0.8 mL ddH_2_O + 0.1 mL 10X TE + 0.1 mL 1 M LiAc), and mixed gently for subsequent use. After that, 5 μL salmon sperm DNA (10 mg/mL), pre‐boiled for 5 min, was added with 5 μg of recombinant plasmids pRB895‐*SAP2* with high *SAP2* expression, and then 0.1 mL aforementioned prepared *C. albicans* SC5314 solution was added. After incubating at room temperature for 30 min, 0.7 mL PLATE Mix (0.8 mL ddH_2_O + 0.1 mL 10X TE + 0.1 mL 1 M LiAc + 0.05 mL inducing expression reagent 5% DMSO) was added. After incubating at room temperature overnight, the fungal solution was subjected to heat shock at 42°C for 3 h, and then centrifuged at 5000 rpm/min for 3 min. The precipitates were resuspended in 0.2 mL sterilized water, and coated onto a selective medium. After culturing at 30°C for 2–3 days, the strain pRB895‐*SAP2*‐SC5314 with high *SAP2* overexpression was obtained. Additionally, the control strain pRB895‐SC5314 with the empty plasmid pRB895 was acquired using the same method.

### 2.4 Screening and Identification of the pRB895‐ *SAP2* ‐SC5314 Strain

pRB895‐*SAP2*‐SC5314 was inoculated onto the selective nutrient deficiency agar medium SD‐ura‐met‐cys, and cultured at 30°C for 2–3 days. After purification for more than three generations, positive clones were obtained, and a fresh single colony was selected and suspended in distilled water to prepare a fungal suspension. Afterward, 500 μL fungal suspension was inoculated into the selective liquid medium, and cultured at 30°C with 200 rpm for 16 h. After centrifugation and washing with PBS thrice, the fungal precipitate was obtained as a template, and the chloramphenicol resistance gene (CMR) was amplified for colony PCR identification. The PCR products of wild‐type *C. albicans* were used as controls, and the size of the amplified fragment was determined. If there were no bands in the wild‐type *C. albicans* and bands appeared in the strain pRB895‐*SAP2*‐SC5314, it was proved that the CMR was transferred into *C. albicans* . According to the CMR sequences provided by Puruting Biotechnology Co., LTD, a primer for *CMR‐1* was designed (pRB895‐SC5314 strain using the primer of *CMR‐2*), and the amplification products of the target bands were sequenced and characterized. The total lengths of *CMR‐1* and *CMR‐2* were 200 and 647 bp, respectively (Table 1). The amplification products (50 μL) and the upper and downstream primers (20 μL of each) were submitted for sequencing, as well as the Snapgene software was used to compare and analyze whether the sequencing results were consistent with the CMR sequences provided by Puruting Biotechnology Co., LTD. The strains with the sequencing results matched correctly were chosen, and stored at −80°C. Additionally, the expression of *SAP2* in the strains of SC5314, pRB895‐SC5314, and pRB895‐*SAP2*‐SC5314 cells was determined using quantitative real‐time reverse transcription polymerase chain reaction (qRT‐PCR).

### 2.5 qRT‐PCR

The strains of SC5314, pRB895‐SC5314, and pRB895‐*SAP2*‐SC5314 were inoculated into the selective nutrient deficiency agar medium SD‐ura‐met‐cys (50 mL), and cultured at 30°C with 200 rpm/min for 16 h. After centrifugation and washing thrice with PBS, fungal precipitates were obtained for RNA isolation using an RNA extraction assay (Shanghai Bioengineering Co. Ltd., Shanghai, China). Total RNA was reverse transcribed into cDNA using the PrimeScript RT Master Mix assay kit (Takara, Dalian, China) according to the manufacturer's instructions. The volume of qRT‐PCR reaction was 20 μL, including 0.8 μL forward primer, 0.8 μL reverse primer, 2 μL cDNA, 6.4 μL DEPC water, and 10 μL 2X TB Green Premix Ex Taq II (Takara). The reaction was initiated at 95°C for 30 s, followed by a total of 40 cycles of 95°C for 5 s, and 60°C for 30 s. The sequences of all primers are listed in Table 1, with *ACT1* serving as the internal gene. All primers used in the qRT‐PCR were validated using 1% agarose gel electrophoresis. The strain SC5314 was used as the control group, ΔΔCT = (CT value of target genes—CT value of internal gene)_experimental group_—(CT value of target genes—CT value of internal gene)_control group_, and the relative expression of related genes was calculated by the 2^−ΔΔCT^ method.

### 2.6 Molecular Docking Prediction

The canonical SMILES of magnolol (PubChem CID: 72300) were obtained from the PubChem database (accession date: 2005‐03‐26, https://pubchem.ncbi.nlm.nih.gov/), and the related traits of magnolol were analyzed using the Swiss ADME platform (accession date: 2017‐03‐03, http://www.swissadme.ch/index.php). The judgment standards were as follows: the score of GI absorption was “High,” while Druglikeness with two or more “Yes” was regarded as good GI absorption. Thereafter, the SDF file of the magnolol 3D structure was downloaded from the PubChem database, and the Open Babel GUI format conversion software was used to convert the SDF file to the mol2 file for further molecular docking. Similarly, a Sap2 protein file (AF‐Q04174‐F1) was obtained from the PDB database (accession date: 1996‐12‐23; https://www.rcsb.org/) and saved as a PDB file. The CB‐DOCK2 platform (accession date: 2021‐01‐04, https://cadd.labshare.cn/cb‐dock2/php/contact.php) was used to perform molecular docking (structure‐based blind docking) and visual analysis of magnolol and Sap2 proteins [20, 21].

### 2.7 Statistical Analysis

Each experiment was repeated three times, and data is expressed as mean ± standard deviation. One‐way analysis of variance using GraphPad Prism 10 software was used for the statistical analysis. *p* < 0.05 was considered statistically significant difference.

## 3 Results

### 3.1 Identification of Plasmids pRB895 and pRB895‐ *SAP2* , as Well as Strains of pRB895‐SC5314 and pRB895‐ *SAP2* ‐SC5314

**FIGURE 1:**
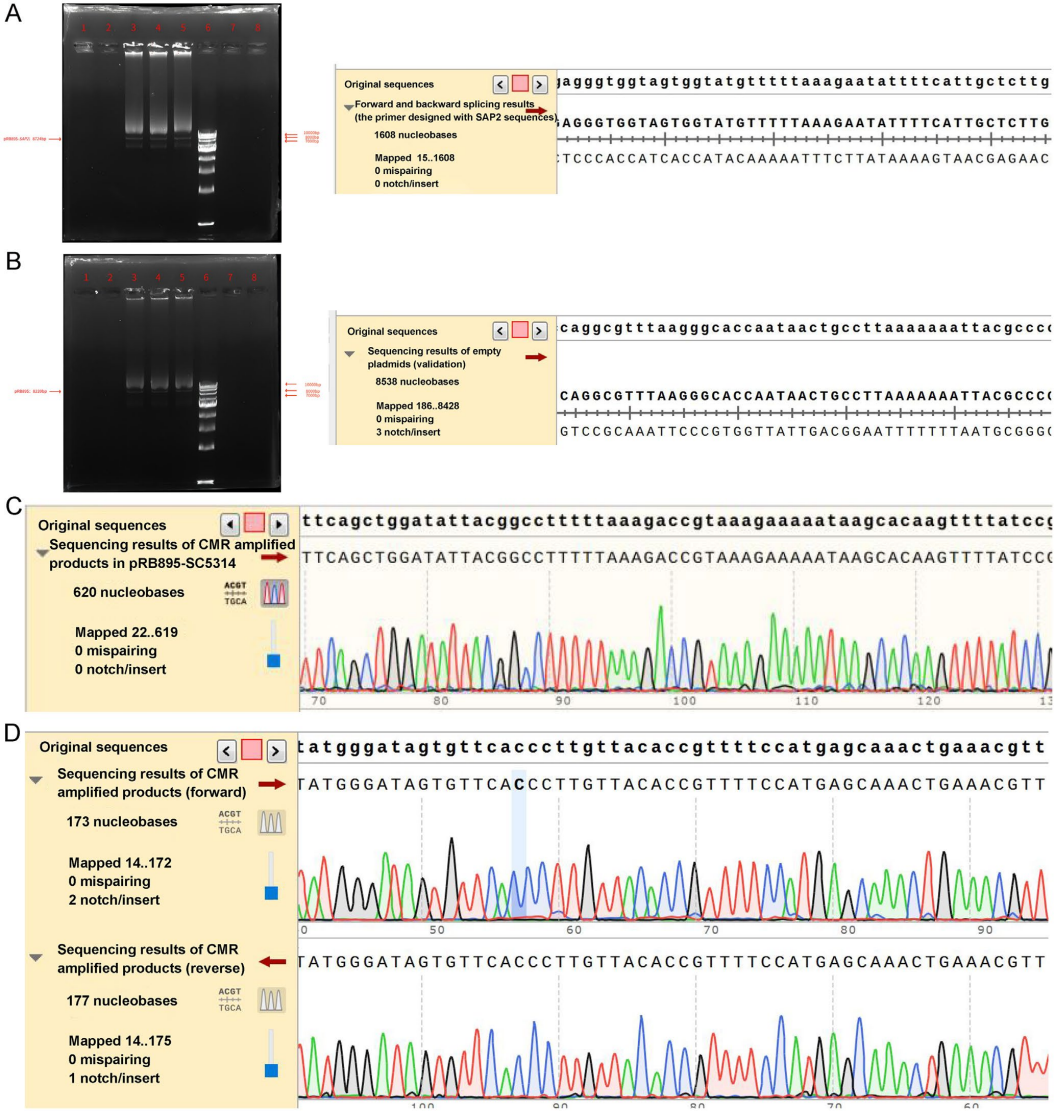
Identification of plasmids pRB895 and pRB895‐*SAP2*, as well as strains pRB895‐SC5314 and pRB895‐*SAP2*‐SC5314. Gel electrophoresis and mapping results of pRB895‐*SAP2* plasmids (A) and empty plasmid pRB895 (B). Sequencing and comparison of pRB895‐SC5314 (C) and pRB895‐*SAP2*‐SC5314 (D).

The gel electrophoresis results for plasmids pRB895 and pRB895‐*SAP2* showed molecular weight of plasmids pRB895 and pRB895‐SAP2 were 8724 bp and 8239 bp, respectively (Figure 1A,B). The sequencing and mapping results of the pRB895‐*SAP2* plasmids showed no mispairing or notches, indicating that the recombinant plasmid pRB895‐*SAP2* with high *SAP2* expression was successfully prepared (Figure 1A). The mapping results of the empty plasmid pRB895 showed no mispairing and three notches, which confirmed that the empty plasmid pRB895 was successfully obtained. Further sequencing and comparison results of the pRB895‐SC5314 strains showed no mispairing and no notches (Figure 1C), and the comparison results of pRB895‐*SAP2*‐SC5314 strains had no mispairing and one notch (Figure 1D). These results imply that *C. albicans* with *SAP2* overexpression (pRB895‐*SAP2*‐SC5314) and *C. albicans* with the empty plasmid pRB895 (pRB895‐SC5314) were successfully constructed.

### 3.2 Validation of all Primers, and the Expression of *SAP2* in the Different Strains

**FIGURE 2:**
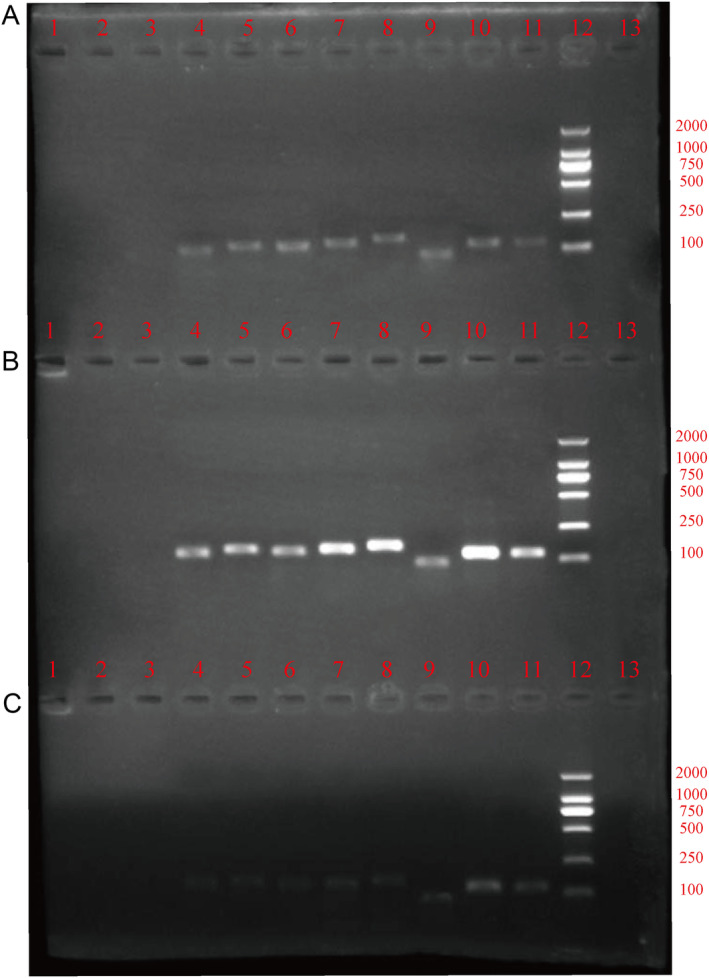
Validation of all primers. Gel electrophoresis results of *HOG1*, *TUP1*, *SAP2*, *PHR1*, *TEC1*, *ALS3*, *ALS1*, and *ACT1* in the strains pRB895‐SAP2‐SC5314 (A), pRB895‐SC5314 (B), and the control strain SC5314 (C). Lanes 4, 5, 6, 7, 8, 9, 10, and 11 represent the primers *HOG1*, *TUP1*, *SAP2*, *PHR1*, *TEC1*, *ALS3*, *ALS1* and *ACT1*, respectively, and lane 12 represents the marker (100–2000 kD).

The gel electrophoresis results of all the primers displayed that in the strains pRB895‐*SAP2*‐SC5314, pRB895‐SC5314, and the control strain SC5314, the primers of *HOG1*, *TUP1*, *SAP2*, *PHR1*, *TEC1*, *ALS3*, *ALS1*, and *ACT1* could be well amplified, and used for further qRT‐PCR determination (Figure 2A–C).

**FIGURE 3:**
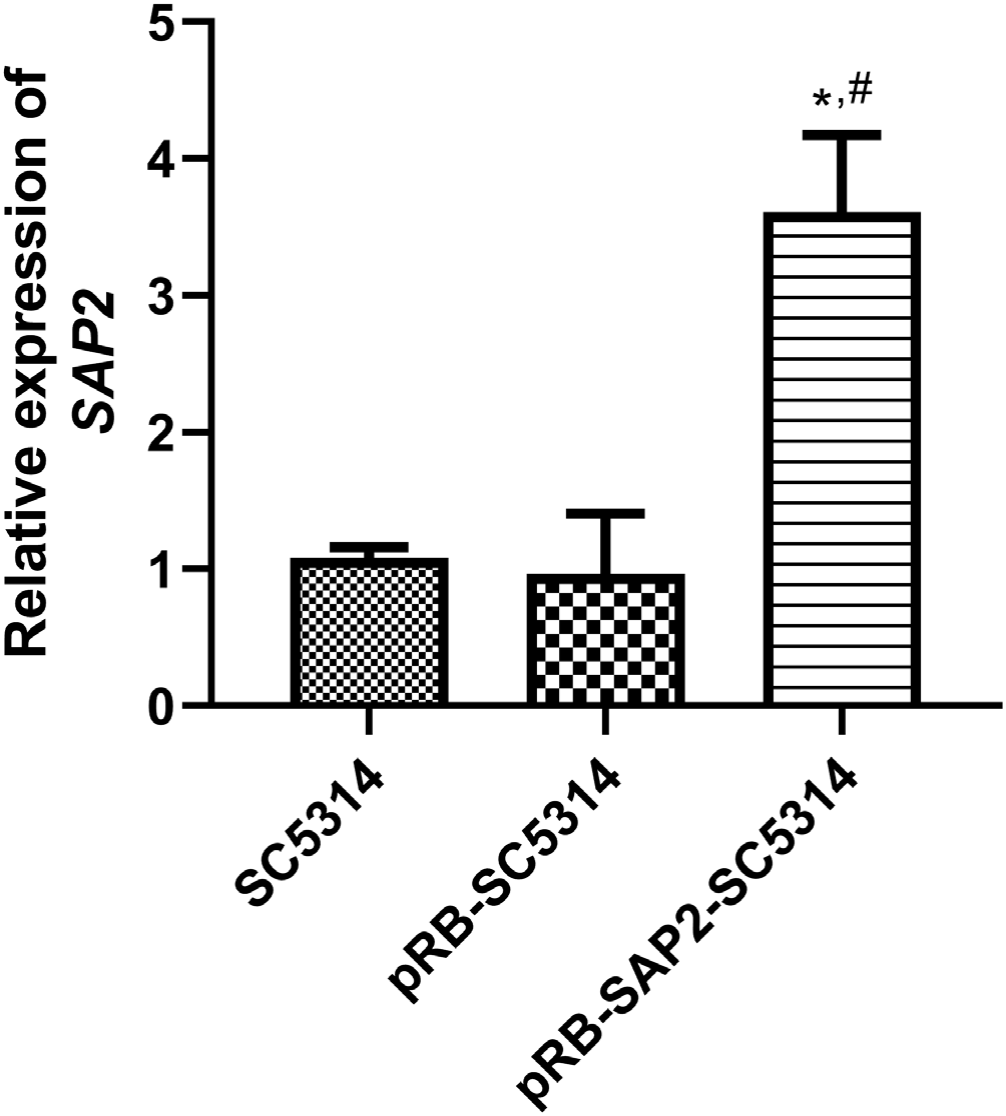
Expression of *SAP2* in SC5314, pRB895‐SC5314, and pRB895‐*SAP2*‐SC5314 strains measured using qRT‐PCR. *p < 0.05 versus SC5314; ^#^ *p* < 0.05 versus pRB895‐SC5314.

To verify the pRB895‐*SAP2*‐SC5314 strains, the expression of *SAP2* was measured in the SC5314, pRB895‐SC5314, and pRB895‐*SAP2*‐SC5314 strains using qRT‐PCR. It was observed that the *SAP2* expression in the strains of SC5314, pRB895‐SC5314, and pRB895‐*SAP2*‐SC5314 were 1.08 ± 0.08, 0.96 ± 0.24, and 3.61 ± 0.36, respectively. These data showed that no significant difference was found in *SAP2* expression between the strains of SC5314 and pRB895‐SC5314 (*p* > 0.05); however, after transfection with pRB895‐*SAP2* plasmids, *SAP2* expression was significantly upregulated in the strains of pRB895‐*SAP2*‐SC5314 (*p* < 0.05, Figure 3), which further suggested that *C. albicans* SC5314 with *SAP2* overexpression was successfully generated and could be used in follow‐up experiments.

### 3.3 Effects of *SAP2* on the Expression of Adhesion‐Related Genes

**FIGURE 4:**
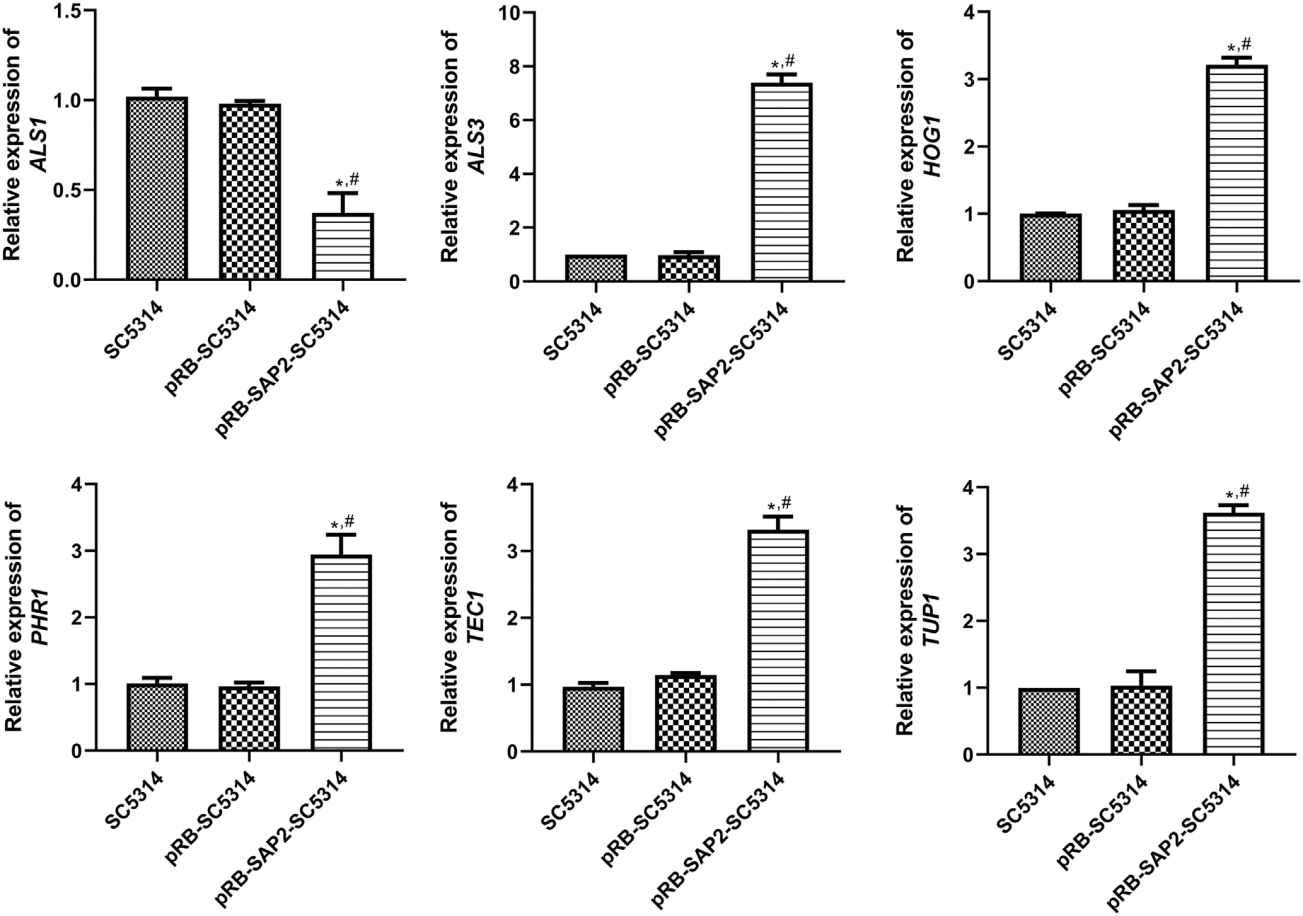
Effects of *SAP2* on the expression of adhesion‐related genes. The expression levels of *ALS1*, *ALS3*, *HOG1*, *PHR1*, *TEC1*, and *TUP1* in the different strains were determined using qRT‐PCR. *p < 0.05 versus SC5314; ^#^ *p* < 0.05 versus pRB895‐SC5314.

To further investigate the effects of *SAP2* on the adhesion ability of *C. albicans* , the expression of adhesion‐related genes, such as *ALS1*, *ALS3*, *HOG1*, *PHR1*, *TEC1*, and *TUP1*, was determined in different strains using qRT‐PCR. It was found that there were no significant differences in the expression of *ALS1*, *ALS3*, *HOG1*, *PHR1*, *TEC1*, or *TUP1* between SC5314 and pRB895‐SC5314 cells (*p* > 0.05; Figure 4). Additionally, compared to the control *C. albicans SC5314*, *ALS1* expression was downregulated, whereas the expression of *ALS3*, *HOG1*, *PHR1*, *TEC1*, and *TUP1* was markedly upregulated in the strains pRB895‐*SAP2*‐SC5314 (*p* < 0.05, Figure 4).

### 3.4 The Interaction Between Magnolol and Sap2 Protein by Molecular Docking

**FIGURE 5:**
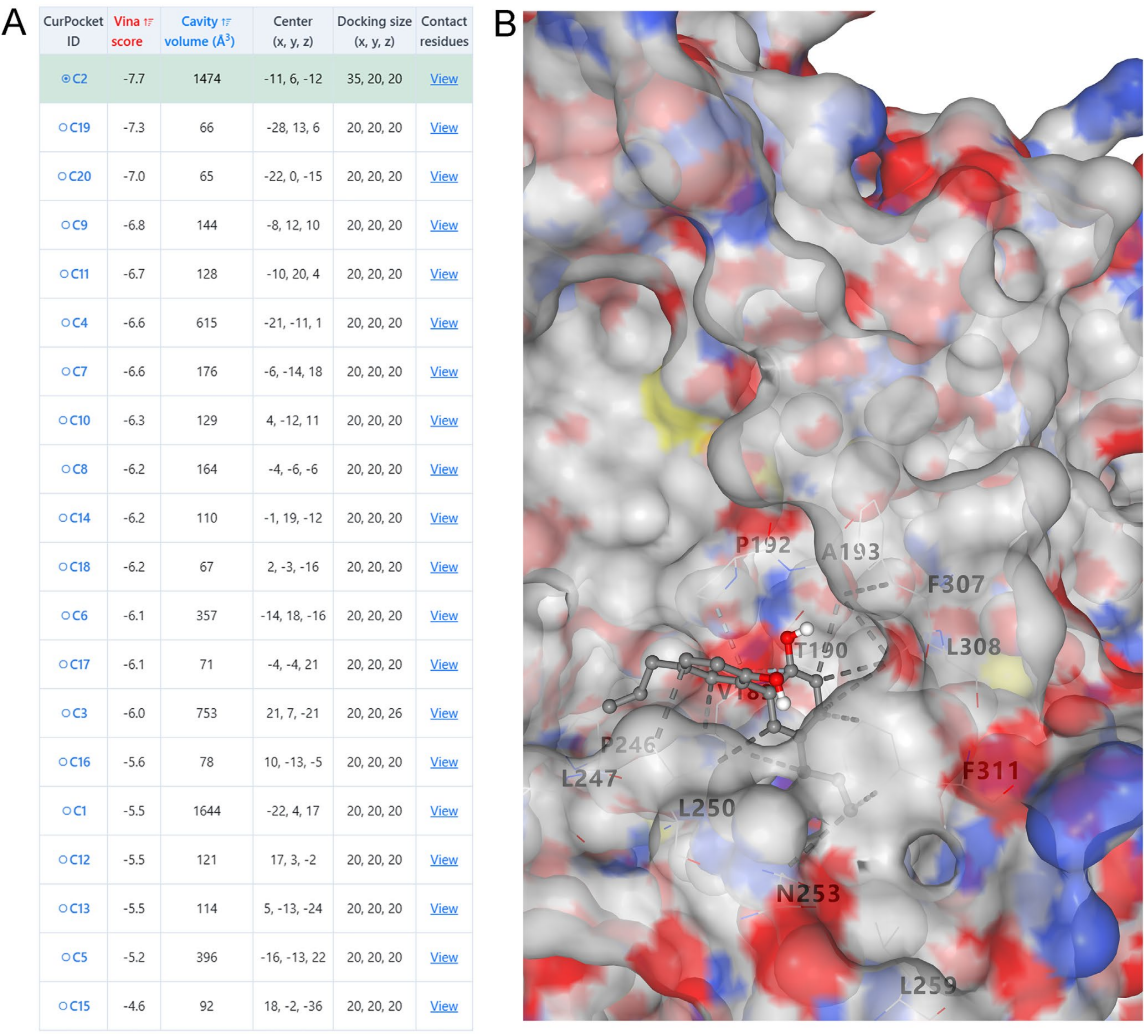
Interaction between magnolol and Sap2 proteins by molecular docking. (A) After 20 molecular docking cycles, the binding energies showed good results. (B) The structure visualization of the optimal coupling results (−8.1 kcal/mol of vina score).

Finally, molecular docking was performed to explore the interaction between magnolol and Sap2. After 20 rounds of molecular docking, the binding energy showed good results (Figure 5A), indicating that magnolol had strong biological activity, which was closely related to the hydrogen bonding between magnolol and Sap2. The optimal result of magnolol docking with Sap2 protein was −8.1 kcal/mol of vina score. Structural visualization of the optimal coupling results showed that magnolol bound to 7 amino acid residues of the Sap2 protein, including F311 (Phe), V189 (Val), L250 (Leu), T190 (Thr), P192 (Pro), A193 (Ala), and L308 (Leu), and 12 hydrogen bonds were formed (Figure 5B).

## 4 Discussion

The completion of genome sequencing of *C. albicans* SC5314 has facilitated the study of its gene function [22]. *C. albicans* is generally a diploid organism; therefore, it is difficult to construct a strain with gene overexpression or knockdown. Previous studies have been limited to determining *SAP2* mRNA expression levels in *C. albicans* strains isolated from the clinical specimens of patients using RT‐qPCR [9, 11]. In this study, a *C. albicans* strain with *SAP2* overexpression (pRB895‐*SAP2*‐SC5314) was successfully prepared by transfection with recombinant pRB895‐*SAP2* plasmids. The strain of pRB895‐*SAP2*‐SC5314 not only retained the physicochemical properties of *C. albicans* SC5314, but also enhances the expression of *SAP2* [23].

Common methods used for gene transformation of *C. albicans* include electroconversion [24], lithium acetate conversion [25], and CRISPR‐Cas9 [26]. Based on the experimental results reported by Bernardi et al. [27], we chose an improved lithium acetate conversion method. The improved lithium acetate conversion method has been shown to significantly improve the plasmid conversion rate of *C. albicans* by adjusting the DMSO chemical treatment and heat shock time based on the traditional lithium acetate conversion method [28]. In the current study, 5% DMSO treatment and heat shock at 42°C for 3 h were selected. DMSO contains sulfur‐oxygen bonds in its molecular structure, which can form hydrogen bonds with water molecules. Additionally, DMSO has non‐polar parts, thus exhibiting good water and lipid solubility as well as high penetration and transport capacity, which can enhance the permeability of cell membranes and facilitate the entry of plasmid DNA into cells [29]. Heat shock also plays an important role in the transformation mechanism, and heat shock proteins act as molecular chaperones that protect structural proteins and enzymes under pressure [30]. Changes in specific proteins induced by heat shock may affect plasmid DNA [31]. Because of the thick cell wall of *C. albicans* , an increase in heat shock time is beneficial for plasmid DNA to penetrate the cell wall and membrane of *C. albicans* , thereby improving the conversion rate. The precise mechanisms of conversion are still unclear [25]; therefore, it is necessary to continue adjusting the conditions for exploration to further increase the conversion efficiency.

The recombinant plasmid pRB895‐*SAP2* with *SAP2* higher expression was transformed into *C. albicans* SC5314 using an improved lithium acetate conversion method. However, there is a high probability of negative converters; therefore, colony PCR products must be verified by agarose gel electrophoresis and sequencing. Simultaneously, the results of qRT‐PCR showed that *SAP2* expression in the positive converters was higher than that in the control group, which fully validated the successful construction of the pRB895‐*SAP2*‐SC5314 strain.

*C. albicans* can form different cell types in the host, including yeast, mycelia, and pseudomycelia, which are closely related to its adhesion ability and virulence of *C. albicans* [32, 33]. The core of the adhesion process involves the expression of adhesion‐related genes. We found that *SAP2* overexpression downregulated *ALS1* expression and upregulated the expression of *ALS3*, *TEC1*, *HOG1*, *PHR1*, and *TUP1* in *C. albicans* . *ALS1* and *ALS3* are adhesin genes in *C. albicans* , and their encoded proteins specifically bind to host receptors and directly participate in host cell adhesion and biofilm formation [34]. In *C. albicans* with *SAP2* overexpression, upregulation of *ALS3* indicated that the strain may have higher colonization and invasion abilities, whereas downregulation of *ALS1* may imply a negative feedback mechanism between different adhesion genes. *TEC1* is a transcription factor associated with mycelial and biofilm formation in *C. albicans* that can activate the expression of *ALS1* and *ALS3* [35]. The higher expression of *TEC1* in *C. albicans* with *SAP2* overexpression suggests that *SAP2* overexpression might promote the morphological transformation of *C. albicans* . The MAPK signal transduction pathway is one of the pathways involved in the morphological transformation of *C. albicans* [36]. The core gene of the MAPK signaling pathway is *HOG1*, which is generally activated during stress response [37]. Higher expression of *HOG1* suggests that *C. albicans* may respond to environmental stress, and its pathogenic ability may be enhanced. *PHR1* interacts with other regulatory genes and signaling pathways in response to changes in environmental pH to jointly regulate cell wall assembly, stability, and morphological transformation in *C. albicans* [38]. The elevated expression of *PHR1* may be associated with cell wall remodeling and mycelial morphology maintenance, which helps *C. albicans* maintain its stability and invasion ability under different pH conditions [39]. *TUP1* is an important transcriptional suppressor. In *C. albicans* , *TUP1* can inhibit the expression of multiple genes by forming complexes with other proteins; therefore, we hypothesized that the enhanced expression of *TUP1* may play a balancing role in the overall regulation of gene expression and prevent the overexpression of certain genes [40]. Taken together, it can be inferred that *SAP2* overexpression may enhance the ability of adhesion, invasion, and mycelial morphologic transformation in *C. albicans* via regulating the expression of *ALS1*, *ALS3*, *TEC1*, *HOG1*, *PHR1*, and *TUP1*. However, the specific roles of these genes in pathogenicity of *C. albicans* are warranted to be studied in the future.

An early study showed that magnolol could suppress the efflux of azoles from *C. albicans* and produce synergistic effects with fluconazole by competing with the ATP‐binding cassette transporter Cdr1p substrate [41]. Another study employed RNA sequencing to illustrate that magnolol at a drug concentration of 160 mg/mL could significantly downregulate the expression of genes related to the pathogenicity of *C. albicans* , including *SAP2*, while weakening the adhesion ability of *C. albicans* [42]. These results suggest a close correlation between magnolol and Sap2. Furthermore, Al Mousa et al. [43] used molecular docking analysis to show that rutin, a potent inhibitor, has a high affinity (−12.35 kcal/mol) for Sap2 in *C. albicans* . Another study performed molecular docking to reveal strong interactions between compound 11a and Sap2 from *C. albicans* [44]. Therefore, we predicted the interaction pattern between magnolol and Sap2 proteins based on computer‐aided molecular docking technology. A lower Vina score indicated a higher affinity for ligand and receptor binding, indicating more stable binding between the two materials [45]. Our data showed that the optimal docking result for magnolol and Sap2 protein was −8.1 kcal/mol of vina score, suggesting that magnolol may act as a Sap2 inhibitor to affect the adhesion of *C. albicans* . However, this hypothesis should be verified in subsequent experiments. At the same time, the coordinate axes (x, y, z) behind the Vina score usually represent the optimal position and conformation of the ligand in the molecular docking model, which helps to understand the specific localization of the ligand in the receptor pocket [46]. This discovery has important implications for the exploration and design of Sap2 inhibitors as effective antibacterial agents for the development of new‐generation antifungal agents or combination therapies.

## 5 Conclusion

We successfully established a *C. albicans* strain with *SAP2* overexpression (pRB895‐*SAP2*‐SC5314) using an improved lithium acetate conversion method. *SAP2* overexpression enhances the pathogenicity of *C. albicans* by regulating the expression of adhesion‐related genes. Additionally, magnolol, a Sap2 inhibitor, may be an effective drug for treating *C. albicans* infections. Our findings provide a new perspective for a deeper understanding of the multilevel regulatory role of *SAP2* in *C. albicans* infection and lay a foundation for *SAP2* and magnolol as novel targets and drugs for the treatment of *C. albicans* infection.

## Author Contributions

Lan Xue and Wenli Feng designed the research. Lan Xue, Lu Yang, Bingqian Zhao, Jing Yang, and Yan Ma did the experiment and obtained the data. Lan Xue and Wenli Feng analysed and explained the data. Lan Xue drafted the manuscript, and Wenli Feng revised. All authors have read and approved the final version.

## Conflicts of Interest

The authors declare no conflicts of interest.

## Data Availability

The data that support the findings of this study are available from the corresponding author upon reasonable request.
